# The RNA m^6^A demethylase ALKBH5 drives emergency granulopoiesis and neutrophil mobilization by upregulating G-CSFR expression

**DOI:** 10.1038/s41423-023-01115-9

**Published:** 2023-12-20

**Authors:** Yang Liu, Renjie Song, Zhike Lu, Lu Zhao, Xinyi Zhan, Yini Li, Xuetao Cao

**Affiliations:** 1 Department of Immunology, Center for Immunotherapy, Institute of Basic Medical Sciences, Peking Union Medical College, Chinese Academy of Medical Sciences, Beijing, China; 2 Suzhou Institute of Systems Medicine, Chinese Academy of Medical Sciences, Suzhou, China; 3 Frontier Research Center for Cell Response, Institute of Immunology, College of Life Sciences, Nankai University, Tianjin, China; 4 School of Life Sciences, Westlake University, Hangzhou, China

**Keywords:** Emergency granulopoiesis, Neutrophil mobilization, ALKBH5, m^6^A RNA modification, G-CSF receptor, Neutrophils, Epigenetics in immune cells

## Abstract

Emergency granulopoiesis and neutrophil mobilization that can be triggered by granulocyte colony-stimulating factor (G-CSF) through its receptor G-CSFR are essential for antibacterial innate defense. However, the epigenetic modifiers crucial for intrinsically regulating G-CSFR expression and the antibacterial response of neutrophils remain largely unclear. *N*^6^-methyladenosine (m^6^A) RNA modification and the related demethylase alkB homolog 5 (ALKBH5) are key epigenetic regulators of immunity and inflammation, but their roles in neutrophil production and mobilization are still unknown. We used cecal ligation and puncture (CLP)-induced polymicrobial sepsis to model systemic bacterial infection, and we report that ALKBH5 is required for emergency granulopoiesis and neutrophil mobilization. ALKBH5 depletion significantly impaired the production of immature neutrophils in the bone marrow of septic mice. In addition, *Alkbh5*-deficient septic mice exhibited higher retention of mature neutrophils in the bone marrow and defective neutrophil release into the circulation, which led to fewer neutrophils at the infection site than in their wild-type littermates. During bacterial infection, ALKBH5 imprinted production- and mobilization-promoting transcriptome signatures in both mouse and human neutrophils. Mechanistically, ALKBH5 erased m^6^A methylation on the *CSF3R* mRNA to increase the mRNA stability and protein expression of G-CSFR, consequently upregulating cell surface G-CSFR expression and downstream STAT3 signaling in neutrophils. The RIP-qPCR results confirmed the direct binding of ALKBH5 to the *CSF3R* mRNA, and the binding strength declined upon bacterial infection, accounting for the decrease in G-CSFR expression on bacteria-infected neutrophils. Considering these results collectively, we define a new role of ALKBH5 in intrinsically driving neutrophil production and mobilization through m^6^A demethylation-dependent posttranscriptional regulation, indicating that m^6^A RNA modification in neutrophils is a potential target for treating bacterial infections and neutropenia.

## Introduction

Neutrophils, one of the most essential populations of innate immune cells, serve as the first line of host defense against invading pathogens, including bacteria [1]. Under physiological conditions, neutrophils are produced and maintained in the bone marrow via normal granulopoiesis. During bacterial infection or under inflammatory conditions, the hematopoietic system utilizes the following four sequential major processes to rapidly boost the innate neutrophil response: step 1) normal granulopoiesis is switched to a protective program, termed “emergency granulopoiesis”, to initiate and accelerate the de novo generation of neutrophils in the bone marrow; step 2) large numbers of neutrophils are mobilized from the bone marrow into the peripheral blood; step 3) circulating neutrophils migrate to distal sites of infection; and step 4) neutrophils perform effector functions such as antibacterial functions [2–4]. In poorly controlled and systemic bacterial infections, neutrophils are in high demand and need to be continuously generated for host defense due to their short lifespan, substantial depletion and death [5, 6]. Therefore, emergency granulopoiesis and neutrophil mobilization are two critical steps that contribute to augmenting neutrophil expansion and the innate immune defense for protecting the host against bacterial infections. Moreover, neutropenia has been widely observed in chemotherapy patients and is associated with a higher risk of clinical disorders [7, 8]. Severe infections in patients with neutropenia are usually fatal [9–11], highlighting the need for a deeper understanding of neutrophil production and mobilization.

Emergency granulopoiesis and neutrophil mobilization can be governed by various cellular and molecular mediators, including the sensing of pathogens by Toll-like receptor pathways in hematopoietic and nonhematopoietic cells [3, 12, 13]; the secretion of extracellular signaling molecules such as growth factors, cytokines, and chemokines [3, 14–19]; and the activation of several transcription factors [20–24]. For instance, activation of CCAAT/enhancer-binding proteins (C/EBPs), especially C/EBP-α and C/EBP-β, is key for inducing steady-state and emergency granulopoiesis, respectively [22, 23]. The cell surface granulocyte colony-stimulating factor receptor (G-CSFR), after activation by its ligand G-CSF, plays the primary role in initiating granulopoiesis and neutrophil mobilization [25–27]. Previous studies have shown that mice with genetic ablation of G-CSFR exhibit impaired granulopoiesis and reduced neutrophil numbers during bacterial infection [25, 27, 28]. G-CSFR mutations and defective G-CSFR signaling have been implicated in several clinical diseases related to disabled granulopoiesis, such as neutropenia—particularly severe congenital neutropenia and chemotherapy-induced febrile neutropenia [29, 30]. Hence, precise control of G-CSFR expression is crucial for neutrophil production and the response to bacterial infection. G-CSFR expression can be transcriptionally regulated. For instance, the T-cell factor/lymphoid enhancer-binding factor (TCF/LEF) transcription factors promote normal and emergency granulopoiesis by directly interacting with the *CSF3R* promoter and enhancer regions to increase G-CSFR expression [20]. However, the role of posttranscriptional regulation mechanisms, such as epigenetic modifications, in controlling G-CSFR expression for neutrophil production and mobilization, remains largely unexplored.

Cell type-specific gene and protein expression patterns, which can be regulated at multiple levels, including the transcriptional and posttranscriptional levels, endow immune cells with their unique phenotypes and specific responses to infections [31–33]. *N*^6^-methyladenosine (m^6^A) RNA modification, the most abundant and common epigenetic modification on mammalian mRNA, has been implicated in various physiological and pathological processes through modulation of RNA metabolism [34]. m^6^A RNA modification and its modifiers can regulate innate immune cell functions. For example, methyltransferase 3, *N*^6^-adenosine-methyltransferase complex catalytic subunit (METTL3)-mediated m^6^A RNA methylation promotes the homeostasis and tumor immunosurveillance function of natural killer cells by increasing the protein expression and activity of SHP-2 [35]. In addition, METTL3 enhances the phenotypic and functional maturation of dendritic cells (DCs) by mediating m^6^A methylation on the *Cd40*, *Cd80*, and *Tirap* mRNAs to enhance their translation [36]. However, whether m^6^A RNA modification can intrinsically regulate neutrophil production and mobilization, especially during bacterial infection, is unclear, and the responsible m^6^A modification enzyme is unknown.

ALKBH5 is an RNA demethylase responsible for removing m^6^A. Our previous study revealed that ALKBH5-mediated m^6^A RNA demethylation rewires cellular metabolism in macrophages to affect host‒virus interactions [37]. Recently, we showed that nuclear ALKBH5 is required for bacterial clearance and mouse survival by empowering neutrophils with a robust ability to respond to chemokine signals and migrate in order to facilitate the accumulation of circulating neutrophils at infection sites, the third step we described above, at the late stage of bacterial infection [38]. During neuroinflammation, T-cell-specific ALKBH5 deletion results in decreased neutrophil recruitment to the central nervous system (CNS) of mice due to the reduced C-X-C motif chemokine ligand 2 (CXCL2) expression in *Alkbh5*-deficient CD4^+^ T cells in the CNS [39]. However, the roles of ALKBH5 and m^6^A RNA modification in neutrophil production and mobilization, the first and second steps we described above, that occur in the bone marrow during the early antibacterial innate response are still unknown. In this study, we demonstrated that ALKBH5 is required for the initiation of emergency granulopoiesis and promotion of neutrophil mobilization in response to bacterial infection by increasing the mRNA stability and protein expression of the G-CSF receptor in both mouse and human neutrophils. Our findings revealed the new role of ALKBH5 and m^6^A RNA demethylation in neutrophil immunobiology and the antibacterial innate defense, providing insight into and potential intervention strategies for neutropenia or neutrophil-associated disorders.

## Results

### ALKBH5 is required for the initiation of emergency granulopoiesis in vivo

**Fig. 1:**
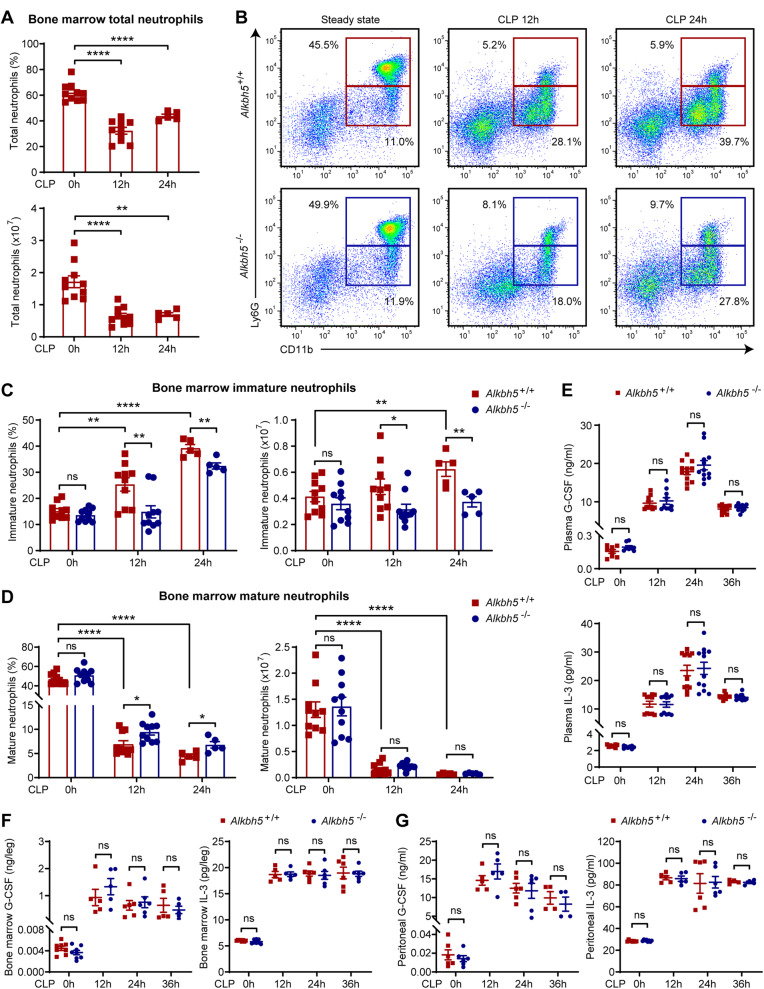
Deficiency of ALKBH5 impairs emergency granulopoiesis in septic mice. **A** Frequencies (top) and numbers (bottom) of total neutrophils (CD11b^+^ Ly6G^+^) in the bone marrow of wild-type (WT) mice at steady state or at the indicated time points after CLP (*n* = 5 or 10). **B** Representative FACS profile showing CD11b^+^ Ly6G^high^ mature and CD11b^+^ Ly6G^low^ immature neutrophils in the bone marrow of *Alkbh5*-deficient mice and their WT littermates in the steady state or at the indicated time points after CLP. **C**, **D** Frequencies and numbers of immature (**C**) and mature (**D**) bone marrow neutrophils as in (**B**) (*n* = 5 or 10). **E**–**G** Concentrations of G-CSF and IL-3 measured by ELISA in the plasma (**E**), bone marrow (**F**), and peritoneal lavage fluid (**G**) of *Alkbh5*-deficient mice and their WT littermates in the steady state or at the indicated time points after CLP (*n* = 4–12). All data are the mean ± SEM of biologically independent samples. The data are representative of 5 or 10 independent experiments with similar results (**B**). Two-tailed unpaired Student’s *t*-test (**A**, **C**–**G**). *P < 0.05; **P < 0.01; ****P < 0.0001; ns not significant

Given the crucial role of neutrophil production in the host antibacterial defense, we investigated whether ALKBH5 might influence granulopoiesis in vivo by performing cecal ligation and puncture (CLP) to establish a well-recognized polymicrobial sepsis model with severe systemic infection that closely resembles clinical sepsis, on *Alkbh5*-deficient mice and their wild-type (WT) littermates. As expected, we observed reductions in the percentage and number of total neutrophils (CD11b^+^ Ly6G^+^) in the bone marrow of WT mice upon CLP-induced sepsis (Fig. 1A and Supplementary Fig. 1A, B). Emergency granulopoiesis characteristically features a reduction in mature bone marrow neutrophils (CD11b^+^ Ly6G^high^) paralleled by an increase in immature bone marrow neutrophils (CD11b^+^ Ly6G^low^) in terms of both the relative percentage and absolute number [12, 20]. Consistently, WT mice in the sepsis model exhibited an increase in immature neutrophils and a significant decline in mature neutrophils in bone marrow, representing the typical hallmarks of emergency granulopoiesis, when compared with unchallenged WT mice (Fig. 1B–D). In the physiological steady state, there was no difference in the percentage or number of bone marrow neutrophils between *Alkbh5*-deficient mice and their WT littermates (Fig. 1B–D), suggesting that ALKBH5 has no impact on normal granulopoiesis. However, a significant reduction in immature neutrophils in the bone marrow was observed in *Alkbh5*-deficient mice in the sepsis model beginning 12 h after CLP, the early stage of bacterial infection (Fig. 1B, C), indicating that a severe defect in emergency granulopoiesis results from ALKBH5 deletion.

G-CSF and interleukin-3 (IL-3) are two major growth factors that stimulate normal granulopoiesis and emergency granulopoiesis during infection [12, 15, 40]. Interestingly, the levels of G-CSF and IL-3 in the plasma (Fig. 1E), bone marrow (Fig. 1F), and peritoneal lavage fluid (Fig. 1G) were comparable between *Alkbh5*-deficient mice and their WT littermates in the steady state and during sepsis, excluding the possibility that the reduced ability to initiate emergency granulopoiesis was due to the altered production of both growth factors in *Alkbh5*-deficient mice. Altogether, these findings indicate that ALKBH5 is required for the initiation of emergency granulopoiesis during the host response to bacterial infection.

### ALKBH5 promotes neutrophil mobilization in response to bacterial infection

**Fig. 2:**
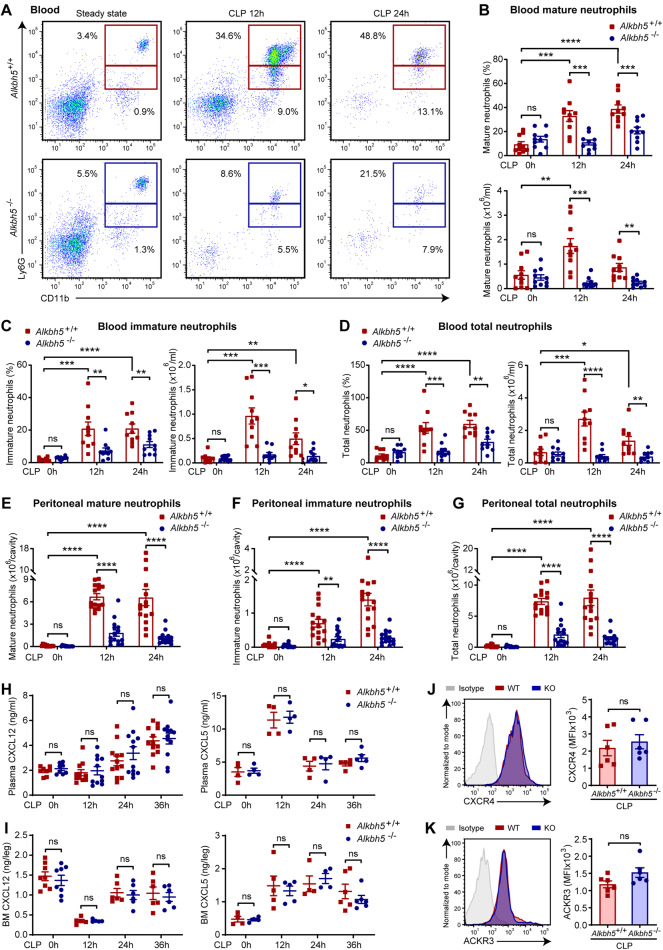
Neutrophil mobilization is disabled in *Alkbh5*-deficient mice during bacterial infection. **A** Representative FACS profile showing CD11b^+^ Ly6G^high^ mature and CD11b^+^ Ly6G^low^ immature neutrophils in the blood of *Alkbh5*-deficient mice and their WT littermates in the steady state or at the indicated time points after CLP. **B**–**D** Frequencies and numbers of mature (**B**), immature (**C**), and CD11b^+^ Ly6G^+^ total (**D**) circulating neutrophils as in (**A**) (*n* = 10). **E**–**G** Numbers of mature (**E**), immature (**F**), and total (**G**) neutrophils in the peritoneal cavity of *Alkbh5*-deficient mice and their WT littermates in the steady state or at the indicated time points after CLP (*n* = 12 or 15). **H**, **I** Concentrations of CXCL12 and CXCL5 measured by ELISA in the plasma (**H**) and bone marrow (BM, **I**) of *Alkbh5*-deficient mice and their WT littermates in the steady state or at the indicated time points after CLP (*n* = 4–12). **J**, **K** FACS analyses of the protein levels of CXCR4 (**J**) and ACKR3 (**K**) on the surface of BM neutrophils from *Alkbh5*-deficient mice and their WT littermates 24 h after CLP (*n* = 6). MFI mean fluorescence intensity. All data are the mean ± SEM of biologically independent samples. The data are representative of 6 or 10 independent experiments with similar results (**A**, **J** and **K**). Two-tailed unpaired Student’s *t*-test (**B**–**K**). *P < 0.05; **P < 0.01; ***P < 0.001; ****P < 0.0001; ns not significant

In response to infection, the pool of neutrophils that mostly resides in the bone marrow under steady-state conditions can be mobilized into the blood, thus providing a mechanism to rapidly deliver neutrophils to sites of infection for host defense [14, 27]. Interestingly, *Alkbh5*-deficient mice exhibited a higher percentage of mature neutrophils in the bone marrow during sepsis (Fig. 1B, D), suggesting a defect in the egress of neutrophils from the bone marrow to blood in *Alkbh5*-deficient mice. Fluorescence-activated cell sorting (FACS) analyses revealed dramatically enhanced release of mature neutrophils from the bone marrow into the peripheral blood in WT mice during sepsis compared with the steady state, yet this increase was far less profound in *Alkbh5*-deficient mice (Fig. 2A, B and Supplementary Fig. 2A). Moreover, the percentages and absolute counts of immature circulating neutrophils and total circulating neutrophils were markedly decreased in *Alkbh5*-deficient mice compared with their WT littermates 12 h after CLP, i.e., the early stage of sepsis (Fig. 2A, C, D). Accordingly, *Alkbh5*-deficient mice displayed substantially reduced numbers of mature, immature, and total neutrophils in the peritoneal cavity, the infection site, during sepsis (Fig. 2E–G and Supplementary Fig. 2B).

Several chemokines and receptors are involved in orchestrating neutrophil mobilization. For instance, C-X-C motif chemokine ligand 12 (CXCL12, also known as SDF-1) is a master retention signal for neutrophil storage in the bone marrow via interactions with its receptors, i.e., C-X-C motif chemokine receptor 4 (CXCR4) and atypical chemokine receptor 3 (ACKR3, also known as CXCR7) [41, 42]. In addition, C-X-C motif chemokine ligand 5 (CXCL5, also known as CXCL6 or LIX) promotes neutrophil recruitment into the peritoneal cavity to protect mice against abdominal sepsis [43]. Loss of ALKBH5 did not alter the levels of CXCL12 and CXCL5 in the plasma, bone marrow or peritoneal lavage fluid of mice in the steady state or during sepsis (Fig. 2H, I and Supplementary Fig. 3A, B). Moreover, there were comparable cell surface levels of CXCR4 and ACKR3 on neutrophils from septic *Alkbh5*-deficient mice compared to those from their septic WT littermates (Fig. 2J, K). Therefore, we excluded the possibility that the impaired neutrophil mobilization in *Alkbh5*-deficient septic mice was mediated by disruption of these factors.

We then asked whether other immune cells might be involved in the process of ALKBH5-mediated neutrophil production and mobilization. FACS analyses showed no significant differences in the populations of several types of immune cells, including DCs, CD4^+^ T cells and CD8^+^ T cells, between septic *Alkbh5*-deficient mice and their septic WT littermates (Supplementary Figs. 4A, C, D and 5A, C, D). The finding of an increased γδ T cell population in the bone marrow of *Alkbh5*-deficient mice (Supplementary Fig. 4E) was consistent with a previous study showing that ALKBH5 inhibits γδ T-cell development [44]. However, *Alkbh5*-deficient mice displayed no changes in peritoneal γδ T cells but reduced numbers of macrophages at later time points of infection (Supplementary Figs. 4B and 5B, E). Considering these results collectively, we speculated that ALKBH5 might primarily promote neutrophil production and mobilization in an intrinsic manner, which leads to subsequent changes in other immune cell populations to support bacterial clearance.

### ALKBH5 remodels the transcriptional landscape in neutrophils to enable their production and mobilization

**Fig. 3:**
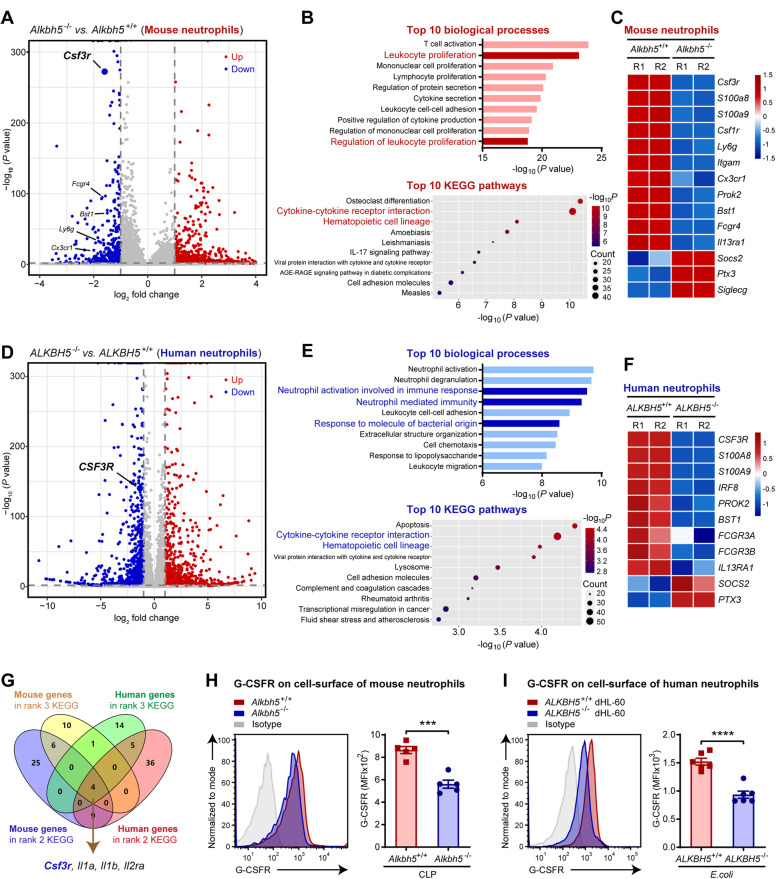
ALKBH5 imprints production- and mobilization-promoting gene signatures in both mouse and human neutrophils. **A** Volcano plot of gene expression profiles in bone marrow neutrophils from *Alkbh5*-deficient mice and their WT littermates 12 h after CLP. The significantly differentially expressed genes (DEGs) upon *Alkbh5* deletion are colored red to indicate upregulation or blue to indicate downregulation. The *x*-axis is limited to genes with |log_2_FC| ≤ 4. Two independent biological replicates. **B** Visualization of the top 10 biological processes (top) and top 10 KEGG pathways (bottom) identified by GO and KEGG enrichment analyses of the significant DEGs upon *Alkbh5* deficiency based on RNA-seq as in (**A**). **C** Heatmap showing the variations in the expression of the DEGs related to neutrophil production and mobilization based on RNA-seq as in (**A**). Two independent biological replicates (R1 and R2). **D** Volcano plot of gene expression profiles in *ALKBH5*-deficient and WT dHL-60 human neutrophils infected with *E. coli*. The significant DEGs upon *ALKBH5* deletion are colored red to indicate upregulation or blue to indicate downregulation. Two independent biological replicates. **E** Visualization of the top 10 biological processes (top) and top 10 KEGG pathways (bottom) identified by GO and KEGG enrichment analyses of the significant DEGs upon *ALKBH5* deficiency based on RNA-seq as in (**D**). **F** Heatmap showing the variations in the expression of the DEGs related to neutrophil production and mobilization based on RNA-seq as in (**D**). Two independent biological replicates (R1 and R2). **G** Integrative analysis of overlapping genes from the indicated KEGG pathways as in (**B**) and (**E**). **H** FACS analyses of the protein level of G-CSFR on the surface of bone marrow neutrophils from *Alkbh5*-deficient mice and their WT littermates 12 h after CLP (*n* = 5). **I** FACS analyses of the G-CSFR protein level on the surface of *ALKBH5*-deficient and WT dHL-60 cells 4 h after *E. coli* infection (*n* = 6). All data are the mean ± SEM of biologically independent samples. The data are representative of 5–6 independent experiments with similar results (**H**, **I**). Two-tailed unpaired Student’s *t*-test (**H**, **I**). ***P < 0.001; ****P < 0.0001

Then, we explored the molecular mechanism of ALKBH5-mediated intrinsic regulation of neutrophils by performing RNA-seq analysis on bone marrow neutrophils isolated from *Alkbh5*-deficient mice and their WT littermates subjected to CLP. RNA-seq analysis showed that loss of ALKBH5 resulted in significant upregulation of 748 genes and downregulation of 371 genes in mouse neutrophils (Fig. 3A). Gene Ontology enrichment analysis of the significantly differentially expressed genes (DEGs) indicated that leukocyte proliferation was one of the most significantly enriched biological processes in *Alkbh5*-deficient bone marrow neutrophils compared to the counterpart WT cells (Fig. 3B, top). Many significant DEGs were also involved in KEGG pathways related to neutrophils, including cytokine‒cytokine receptor interaction and hematopoietic cell lineage (Fig. 3B, bottom). Notably, loss of ALKBH5 substantially decreased the transcript levels of several receptors of cytokines or factors that are critical for neutrophil production and mobilization, such as colony-stimulating factor 3 receptor (*Csf3r*, also known as G-CSFR), C-X3-C motif chemokine receptor 1 (*Cx3cr1*) and Fc receptor, IgG, low-affinity IV (*Fcgr4*) (Fig. 3A, C). Conversely, ALKBH5 deletion increased the mRNA levels of negative regulators of neutrophil production and function, such as suppressor of cytokine signaling 2 (*Socs2*) and pentraxin-related gene (*Ptx3*) (Fig. 3C). The transcript levels of two markers of mouse neutrophils, lymphocyte antigen 6 family member G (*Ly6g*) and integrin alpha M (*Itgam*, also known as *Cd11b*), were reduced in neutrophils from *Alkbh5-*deficient septic mice (Fig. 3C). In addition, the significantly altered expression of these genes in mice was verified via qRT‒PCR (Supplementary Fig. 6A), further confirming the credibility and accuracy of the sequencing data.

As ALKBH5 is highly conserved between mice and humans [45], we then confirmed the ALKBH5-driven transcriptional landscape in human neutrophils by using an in vitro model of differentiated HL-60 neutrophil-like (dHL-60) cells infected with *Escherichia coli* (*E. coli*). RNA-seq analysis of *ALKBH5*-deficient and WT dHL-60 cells showed that ALKBH5 deletion significantly upregulated 1255 genes but downregulated 1035 genes in bacteria-infected human neutrophils (Fig. 3D). Most of the genes that were significantly differentially expressed in *ALKBH5*-deficient dHL-60 cells compared with WT dHL-60 cells were enriched in biological processes associated with neutrophil-mediated immune responses or in pathways related to granulopoiesis and neutrophil mobilization (Fig. 3E, F), and *ALKBH5*-deficient dHL-60 cells exhibited transcriptional signatures similar to those of bone marrow neutrophils isolated from *Alkbh5-*deficient septic mice (Fig. 3B, C). The qRT‒PCR results confirmed that mRNA levels of these genes were significantly down- or upregulated, consistent with the RNA-seq data, in human neutrophils lacking ALKBH5 expression (Supplementary Fig. 6B). These results further highlighted the crucial role of ALKBH5 in enabling neutrophil production and mobilization.

Based on the KEGG pathways with the greatest enrichment, we identified the downstream targets of ALKBH5 by integrative analysis of the overlapping significant DEGs in mouse and human neutrophils (Fig. 3G). Among the four overlapping genes, a particularly interesting one was *Csf3r*, whose transcript level was significantly decreased by ALKBH5 deletion (Fig. 3A, D). *Csf3r* encodes G-CSFR, the central driver of granulopoiesis and neutrophil mobilization [20, 27, 30]. We then determined whether the defective neutrophil production and mobilization in *Alkbh5*-deficient mice result from reduced G-CSFR expression. FACS analyses showed that the protein levels of G-CSFR were indeed lower on the surface of bone marrow neutrophils from *Alkbh5*-deficient septic mice than on those from their WT littermates (Fig. 3H). Consistently, significantly decreased surface G-CSFR expression was observed on *ALKBH5*-deficient dHL-60 human neutrophils upon bacterial infection (Fig. 3I). Together, these findings indicate that ALKBH5 induces a transcriptional program in both mouse and human neutrophils to intrinsically drive emergency granulopoiesis and neutrophil mobilization.

### ALKBH5 upregulates cell surface G-CSFR expression and augments G-CSFR downstream signaling in neutrophils

**Fig. 4:**
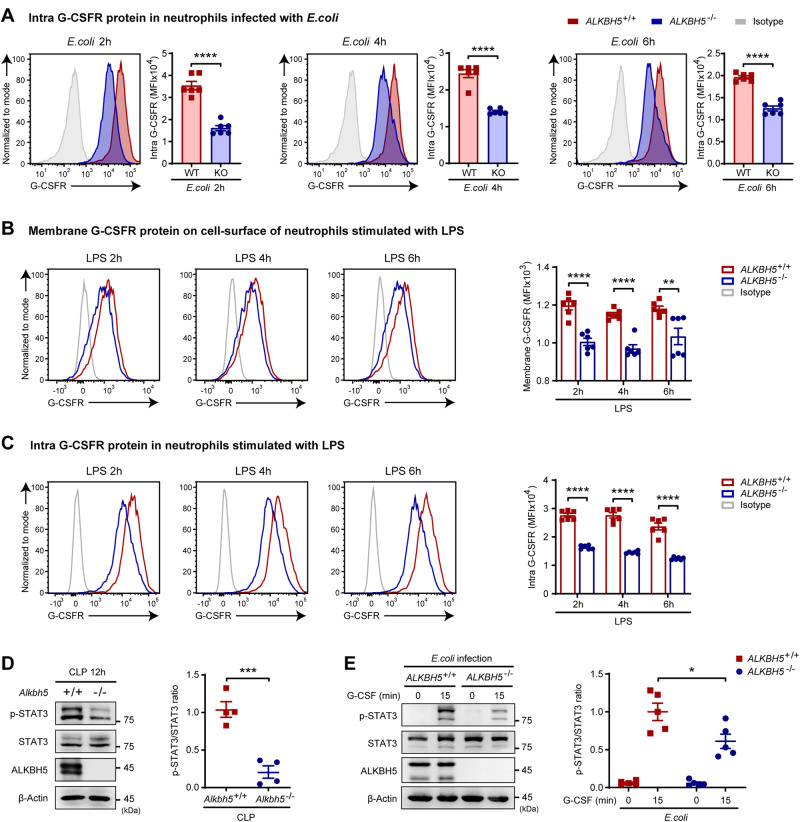
Loss of ALKBH5 impairs G-CSFR signaling in neutrophils during bacterial infection. **A** FACS analyses of total intracellular levels of G-CSFR protein in *ALKBH5*-deficient and WT dHL-60 cells infected with *E. coli* for the indicated times (*n* = 6). **B**, **C** FACS analyses of cell surface (**B**) and total intracellular (**C**) levels of G-CSFR protein in *ALKBH5*-deficient and WT dHL-60 cells stimulated with LPS for the indicated times (*n* = 6). **D** Analyses of STAT3 signaling in bone marrow neutrophils from *Alkbh5*-deficient mice and their WT littermates 12 h after CLP by immunoblotting (left) and integrated density measurement (right, *n* = 4). **E** Analyses of STAT3 signaling in *ALKBH5*-deficient and WT dHL-60 cells infected with *E. coli* and then treated with human G-CSF (100 ng/ml) for the indicated times by immunoblotting (left) and integrated density measurement (right, *n* = 5). The integrated density was determined using the ImageJ program (**D**, **E**). All data are the mean ± SEM of biologically independent samples. The data are representative of 4 or 5 independent experiments with similar results (**D**, **E**). Two-tailed unpaired Student’s *t*-test (**A**–**E**). *P < 0.05; **P < 0.01; ***P < 0.001; ****P < 0.0001

G-CSFR is required for neutrophil responses by driving granulopoiesis and neutrophil mobilization, especially in the antibacterial defense [20, 26, 27]. We therefore tested the effect of ALKBH5 on G-CSFR expression and its downstream signaling. FACS analyses revealed that total intracellular G-CSFR protein expression was substantially reduced in *ALKBH5*-deficient dHL-60 cells during *E. coli* infection (Fig. 4A), consistent with our observation of decreased G-CSFR protein expression on the surface of neutrophils (Fig. 3H, I). During lipopolysaccharide (LPS) stimulation, *ALKBH5*-deficient neutrophils also exhibited significantly reduced protein levels of cell surface and intracellular G-CSFR (Fig. 4B, C). These findings suggest that G-CSFR is the downstream target of ALKBH5 and that its protein expression can be upregulated by ALKBH5.

Upon binding to G-CSF, G-CSFR activates its downstream signaling, especially signal transducer and activator of transcription 3 (STAT3) signaling, to induce neutrophil production and egress from the bone marrow into the blood [17, 20, 21]. Knockdown of G-CSFR suppressed STAT3 activation in neutrophils after G-CSF treatment (Supplementary Fig. 7A, B). Notably, bone marrow neutrophils from *Alkbh5*-deficient septic mice exhibited defective phosphorylation of STAT3 (Fig. 4D). In addition, *ALKBH5*-deficient dHL-60 cells also displayed an attenuated response to G-CSF stimulation, having a lower level of phosphorylated STAT3 (Fig. 4E). These findings imply that ALKBH5 directly upregulates the protein expression of G-CSFR to increase its cell surface level on neutrophils to mediate G-CSFR-STAT3 signaling, thus contributing to emergency granulopoiesis and neutrophil mobilization.

### ALKBH5-mediated m^6^A demethylation enhances the stability of *CSF3R* mRNA

**Fig. 5:**
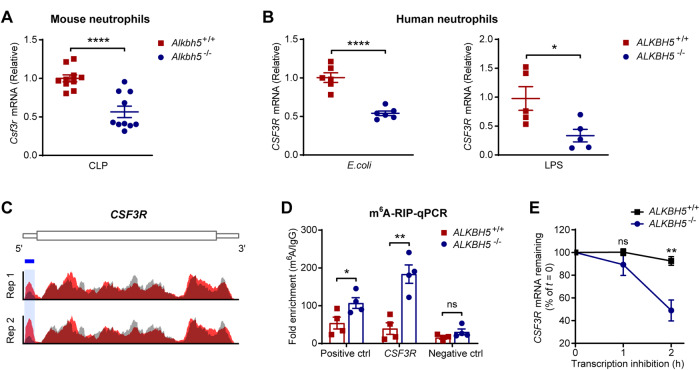
ALKBH5 deficiency increases m^6^A modification of the *CSF3R* mRNA to promote its decay. **A** qRT‒PCR analysis of *Csf3r* mRNA level in bone marrow neutrophils isolated from *Alkbh5*-deficient mice and their WT littermates 12 h after CLP (*n* = 10). **B** qRT‒PCR analysis of *CSF3R* mRNA level in *ALKBH5*-deficient and WT dHL-60 cells infected with *E. coli* (left, *n* = 6) or stimulated with LPS (right, *n* = 5). mRNA level data were normalized to *Gapdh* (**A**) or *GAPDH* (**B**) expression. **C** m^6^A abundance on the *CSF3R* mRNA in dHL-60 cells infected with *E. coli* as indicated by our m^6^A-seq data (GSE201060, NM_000760.4). Two independent biological replicates (Rep 1 and Rep 2). Red, m^6^A-IP; gray, input; blue box, m^6^A peaks. The *y*-axis shows the normalized m^6^A signal along the gene. **D** m^6^A-RIP-qPCR analysis of m^6^A enrichment of the *CSF3R* mRNA in *ALKBH5*-deficient and WT dHL-60 cells infected with *E. coli* for 2 h (*n* = 4). Different regions of human *EEF1A1* mRNA were used as positive and negative controls (ctrl). The data were presented relative to those obtained with IgG. **E** RNA decay assay of *CSF3R* mRNA degradation in *ALKBH5*-deficient and WT dHL-60 cells treated with actinomycin D for the indicated times (*n* = 5). The data were normalized to the *18S* rRNA level, and residual mRNA abundances were normalized to the abundance at *t* = 0 h. All data are the mean ± SEM of biologically independent samples. Two-tailed unpaired Student’s *t*-test (**A**, **B**, **D** and **E**). *P < 0.05; **P < 0.01; ****P < 0.0001; ns not significant

Next, we explored the mechanism through which ALKBH5 upregulates G-CSFR expression. m^6^A modification-related enzymes modulate gene expression by altering m^6^A modifications on their target mRNAs to regulate RNA metabolic processes, such as mRNA stability and translation [34, 36, 46]. The RNA-seq data showed that ALKBH5 deletion significantly decreased the transcript level of *CSF3R* in both mouse and human neutrophils upon bacterial infection (Fig. 3A, D). Consistently, qRT‒PCR showed that the *Csf3r* mRNA level was indeed decreased in bone marrow neutrophils from *Alkbh5*-deficient mice compared with those from their WT littermates during sepsis (Fig. 5A). Moreover, deletion of ALKBH5 significantly decreased the level of *CSF3R* mRNA in dHL-60 human neutrophils upon bacterial infection and LPS stimulation (Fig. 5B).

To determine whether ALKBH5 can regulate the *CSF3R* mRNA level in an m^6^A RNA modification-dependent manner, we analyzed our previous transcriptome-wide m^6^A methylation profiling (m^6^A-seq) data from neutrophils, which exhibited strong correlations between the two biological replicates (Supplementary Fig. 8A). Notably, m^6^A-seq analysis revealed highly enriched and specific m^6^A peaks on the *CSF3R* mRNA in bacteria-infected neutrophils (Fig. 5C). Furthermore, the results of m^6^A RNA immunoprecipitation combined with qRT‒PCR (RIP**-**qPCR) verified that the *CSF3R* mRNA indeed contained m^6^A modifications and that the m^6^A level on the *CSF3R* mRNA was significantly higher in *ALKBH5*-deficient neutrophils than in WT neutrophils (Fig. 5D). Therefore, *CSF3R* transcripts are m^6^A targets directly modulated by ALKBH5 in neutrophils. ALKBH5 acts as an m^6^A demethylase and modulates gene expression by controlling m^6^A modification-mediated mRNA degradation [47–49]. Next, we performed RNA decay assays and confirmed that *ALKBH5* deficiency markedly promoted the degradation of *CSF3R* mRNA in neutrophils after transcriptional inhibition (Fig. 5E). Altogether, these results indicate that ALKBH5 specifically removes m^6^A from the *CSF3R* mRNA to delay its decay, consequently increasing the mRNA and protein expression of G-CSFR in neutrophils.

### Bacterial infection downregulates G-CSFR expression by inhibiting the binding of ALKBH5 to the *CSF3R* mRNA in neutrophils

**Fig. 6:**
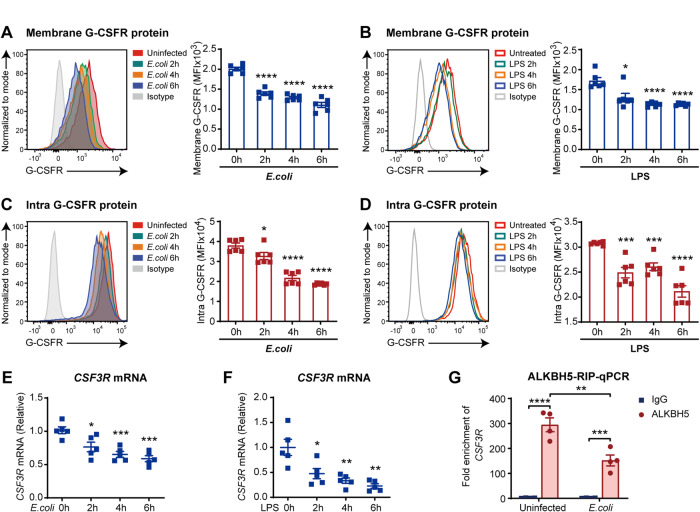
Reduced binding of ALKBH5 to the *CSF3R* mRNA leads to decreased G-CSFR expression in bacteria-infected neutrophils. **A**, **B** FACS analyses of the protein levels of G-CSFR on the surface of WT dHL-60 cells stimulated with *E. coli* (**A**) or LPS (**B**) for the indicated times (*n* = 6). **C**, **D** FACS analyses of total intracellular G-CSFR protein in WT dHL-60 cells stimulated with *E. coli* (**C**) or LPS (**D**) for the indicated times (*n* = 6). **E**, **F** qRT‒PCR analysis of *CSF3R* mRNA level in WT dHL-60 cells stimulated with *E. coli* (**E**) or LPS (**F**) for the indicated times (*n* = 5). **G** RIP-qPCR analysis of ALKBH5 binding to the *CSF3R* mRNA in WT dHL-60 cells without or with *E. coli* infection for 4 h (*n* = 4). The data were presented relative to those obtained with IgG. All data are the mean ± SEM of biologically independent samples. The data are representative of 6 independent experiments with similar results (**A**–**D**). Two-tailed unpaired Student’s *t*-test (**A**–**G**). *P < 0.05; **P < 0.01; ***P < 0.001; ****P < 0.0001

Bacterial infection can trigger septic shock by desensitizing the neutrophil response to G-CSF and impairing granulopoiesis [50, 51]. Therefore, we determined the effect of bacterial infection on G-CSFR expression in our infection model. As speculated, not only the cell surface protein level (Fig. 6A, B) but also the total intracellular protein level (Fig. 6C, D) of G-CSFR was significantly decreased in WT neutrophils upon *E. coli* infection or LPS stimulation. Furthermore, qRT‒PCR showed that *E. coli* infection and LPS stimulation decreased the *CSF3R* mRNA level in WT neutrophils (Fig. 6E, F). Via RIP-qPCR, we confirmed that the ALKBH5 protein exhibited high enrichment and direct binding of its target, the *CSF3R* mRNA, in neutrophils. Interestingly, the amount of ALKBH5 bound to the *CSF3R* mRNA was lower during bacterial infection than under physiological conditions (Fig. 6G). These data indicate the possible pathological significance of reduced binding of ALKBH5 to or its dissociation from the *CSF3R* mRNA for insufficient G-CSFR expression in neutrophils, which makes neutrophils insensitive to G-CSF, resulting in disabled emergency granulopoiesis and neutrophil mobilization in severe infections.

## Discussion

**Fig. 7:**
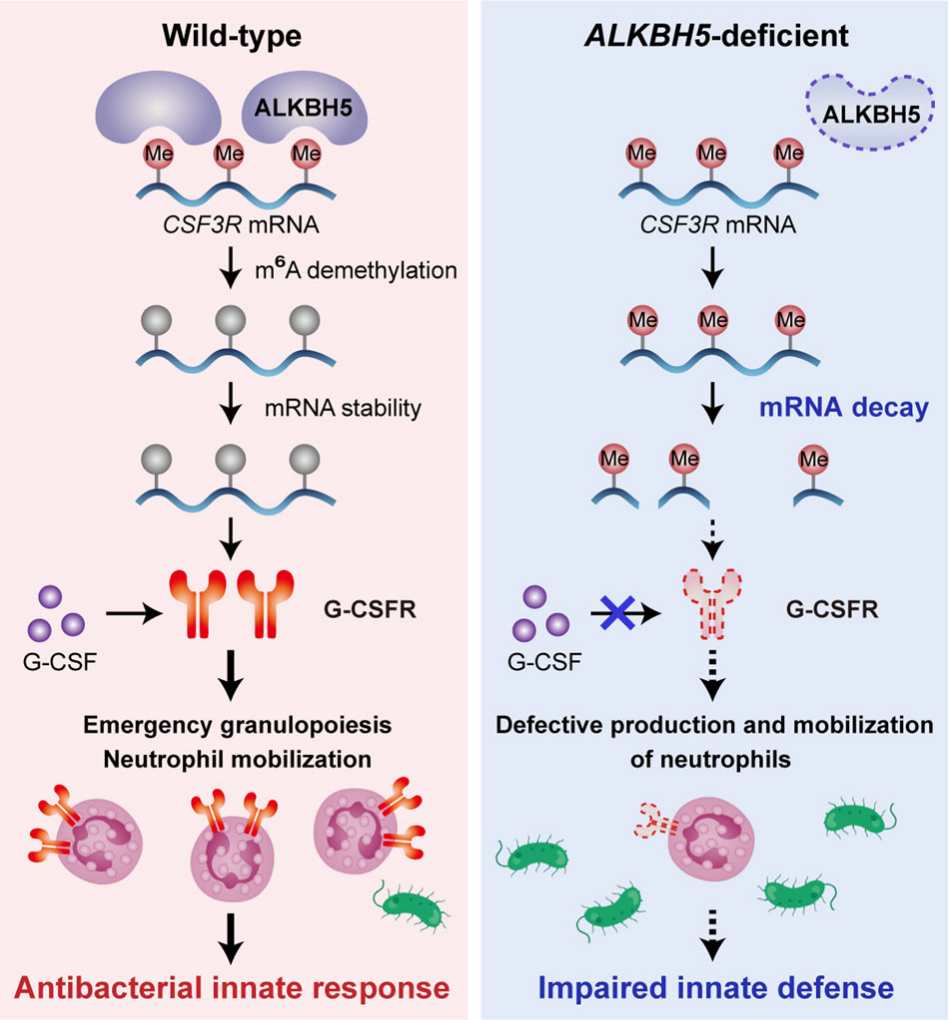
Roles of ALKBH5-mediated m^6^A demethylation in neutrophil production and mobilization through posttranscriptional enhancement of *CSF3R* mRNA stability and upregulation of G-CSFR protein expression. ALKBH5 directly binds to the *CSF3R* mRNA and mediates its m^6^A demethylation to increase the mRNA stability and consequent protein expression of G-CSFR, maintaining the antibacterial innate response (left). Upon ALKBH5 deletion or bacterial infection, disruption of the ALKBH5-*CSF3R* mRNA axis leads to an increased m^6^A level on the *CSF3R* mRNA, resulting in enhanced *CSF3R* mRNA decay and then decreased G-CSFR protein expression, which impairs neutrophil production and mobilization as well as the host innate defense (right). Me, m^6^A RNA modification

The systemic neutrophil response contributes to the host innate defense via multiple steps that involve neutrophil production and mobilization, which were previously known to be controlled by extracellular signals or by modulation of gene expression at the transcriptional level [52]. m^6^A RNA modification and its demethylase ALKBH5 play critical roles in epigenetic regulation of immune cell functions, but their effect on neutrophil production and mobilization is still unknown. In this study, we defined a new cell-intrinsic role of ALKBH5 in allowing emergency granulopoiesis and neutrophil mobilization through upregulation of G-CSFR expression in neutrophils via an m^6^A RNA demethylation-dependent mechanism, providing new insights into the epigenetic regulation of the antibacterial innate defense. In the steady state, ALKBH5 directly binds the *CSF3R* mRNA and mediates m^6^A demethylation to increase the mRNA stability and protein expression of G-CSFR to maintain homeostasis. However, the ALKBH5-*CSF3R* mRNA axis can be disrupted upon ALKBH5 deficiency or bacterial infection, leading to m^6^A modification-induced decay of the *CSF3R* mRNA and a decreased G-CSFR protein level, which impairs neutrophil production and mobilization and results in failure of the host innate defense (Fig. 7).

To date, limited data are available on the effects of intrinsic factors on posttranscriptional regulation of innate immune cell production, especially in the context of host defense. To our knowledge, our findings reveal a previously unknown cell-intrinsic mechanism for emergency granulopoiesis mediated through m^6^A RNA modification-mediated epigenetic regulation. Neutrophils utilize specific transcription factors to shape their development by regulating gene expression at the transcriptional level. In addition to C/EBP-β, other transcription factors, such as Gfi-1 and IRF8, also modulate emergency granulopoiesis [23, 53, 54]. DNA methylation, one kind of epigenetic modification, is dynamic in enhancer elements to alter gene expression during granulopoiesis [55], shedding light on an essential role of epigenetic regulation in determining neutrophil production. However, which epigenetic factor is required for the posttranscriptional regulation of emergency granulopoiesis remains unknown. Recent evidence has identified m^6^A RNA modification as a key epigenetic player in adaptive immune cell development. For example, the m^6^A writer METTL3 maintains naive T cell proliferation and differentiation by increasing mRNA degradation of *SOCS* family members [56]. However, there is little knowledge about the role of m^6^A RNA modification and the related enzymes in innate immune cell production and mobilization during infections. Here, we revealed that m^6^A RNA modification is correlated with impaired neutrophil production and release. Subsequent assays elucidated that m^6^A modification of the *CSF3R* mRNA decreases its stability. Therefore, m^6^A RNA modification acts as an intrinsic negative regulatory factor that posttranscriptionally controls emergency granulopoiesis and neutrophil mobilization during the antibacterial defense.

Neutrophils have been found to play dominant roles in the host innate defense during polymicrobial infections and in a CLP-induced sepsis mouse model [2, 40, 57]. In our study, mice lacking ALKBH5 expression exhibited significantly impaired emergency granulopoiesis and neutrophil mobilization beginning 12 h after CLP, i.e., the early stage of sepsis. Then, a reduction in the macrophage count was observed in *Alkbh5*-deficient mice 24 h after CLP, which might be a consequence of the reduced number of neutrophils at the same sites. Consistent with the previous finding that depletion of ALKBH5 in lymphocytes can result in the specific expansion of γδ T cell precursors [44], our FACS analyses showed an increase in γδ T cells in the bone marrow of *Alkbh5*-deficient septic mice. Considering that γδ T cells develop in the thymus [44] and constitute less than 1% of bone marrow leukocytes 12 h after CLP, they may have only a minor impact on neutrophil production and mobilization, which occur mainly in the bone marrow during sepsis. In addition, ALKBH5 deletion did not affect the γδ T cell distribution in the peritoneal cavity, the major infection site in mice subjected to CLP, implying that γδ T cells are not responsible for the defective antibacterial defense in *Alkbh5*-deficient septic mice. Together, these observations indicate that ALKBH5 preferentially and initially drives neutrophil production and mobilization by increasing the cell surface G-CSFR level on neutrophils, which further enhances the antibacterial innate defense via subsequent minor effects on macrophages, highlighting an intrinsic role of ALKBH5 in directly triggering the neutrophil response.

G-CSFR signaling is key for granulopoiesis and neutrophil mobilization. The membrane G-CSFR level has been reported to be regulated by toxins from pathogenic bacteria or by transcription factors within neutrophils. For instance, the α-toxin produced by the gram-positive anaerobic bacterium *Clostridium perfringens* induces the degradation of neutrophil G-CSFR [50]. Activation of the transcription factors C/EBPepsilon and LEF-1 promotes G-CSFR expression at the transcriptional level [20, 58]. Our present study shows that ALKBH5 upregulates G-CSFR protein expression by directly removing m^6^A from the *CSF3R* mRNA to enhance its stability. In addition to previous works, we reveal for the first time a previously unrecognized mechanism underlying epigenetic regulation of G-CSFR expression in an m^6^A RNA modification-dependent posttranscriptional manner.

During infections, bacteria may evade the host innate defense by exploiting a negative regulator of the immune system and disabling neutrophil responses, a strategy exemplified by the impaired neutrophil production and mobilization caused by epigenetic dysregulation of the ALKBH5-*CSF3R* mRNA axis, as revealed in this study. Indeed, bacterial infection can make neutrophils insensitive to G-CSF, and a decreased cell surface G-CSFR level on bacteria-infected neutrophils has previously been observed [59]. This immune paralysis convinced us that alternative key regulators and new cell-intrinsic mechanisms underlying neutrophil homeostasis remain largely unexplored. Here, we show that G-CSFR downregulation is responsible for impaired neutrophil production and mobilization in septic mice lacking ALKBH5 expression. Furthermore, G-CSFR expression is downregulated in neutrophils upon bacterial infection because of decreased binding of ALKBH5 to the *CSF3R* mRNA. One question remains regarding the mechanisms of the disruption of the ALKBH5-*CSF3R* mRNA interaction in bacteria-infected neutrophils. In addition, further study is needed to investigate the possible effect of ALKBH5 on the proliferation of early granulocytic precursors and their differentiation into mature neutrophils.

Our findings indicate potential applications of the ALKBH5–m^6^A RNA demethylation–G-CSFR axis in rescuing neutropenia or low G-CSFR expression. Failure of granulopoiesis and neutrophil mobilization have been widely observed in patients with pathological conditions, including septic shock, neutropenia, bone marrow transplantation, and chemotherapy-induced myelosuppression, which are associated with high mortality [7, 60–64]. Future investigation of the possible change in the ALKBH5-*CSF3R* mRNA interaction in neutrophils induced by chemotherapy might be helpful in identifying new targets for cancer immunotherapy. Although G-CSF is widely used for the clinical treatment of patients with the above-mentioned conditions, there are still some limitations in the efficacy of clinical G-CSF treatment due to G-CSF refractoriness [65, 66]. For instance, patients who respond poorly to G-CSF because of relatively low expression of G-CSFR have a higher risk of developing severe illness [67], emphasizing the key roles of ALKBH5 and m^6^A RNA demethylation in the positive regulation of G-CSFR expression. Therefore, specific activation or upregulation of the ALKBH5–m^6^A RNA demethylation–G-CSFR axis in neutrophils may restore a protective host innate defense and be a potentially promising strategy to treat neutropenia and bacterial infectious diseases.

## Materials and methods

### Mice

*Alkbh5*-deficient mice on a C57BL/6 background were generated as previously described [37, 68]. Mice were bred and maintained under specific-pathogen-free conditions, and 8- to 10-week-old littermate mice were used. All mouse experiments were performed in accordance with the National Institutes of Health Guide for the Care and Use of Laboratory Animals, with approval of the Animals Care and Use Committees of the Institute of Laboratory Animal Sciences of Chinese Academy of Medical Sciences (ACUC-A01-2020-004).

### Cecal ligation and puncture (CLP)-induced sepsis model

The sepsis mouse model was established as previously described [57]. In detail, the peritoneal cavity was opened after the mouse was anesthetized. The cecum was externalized and ligated at a point approximately 50% of the distance from the ileocecal valve with a nonabsorbable suture to induce mid-grade sepsis in this study. Then, the distal end of the cecum was perforated with a 21G needle, and a small drop of feces was extruded through the puncture. Finally, the cecum was relocated into the peritoneal cavity, and the peritoneum was closed.

### Flow cytometry

Single-cell suspensions were obtained from the bone marrow, peripheral blood, and peritoneal lavage fluid of 8- to 10-week-old *Alkbh5*-deficient mice and their wild-type (WT) littermates and were then stained with fluorophore-conjugated antibodies as described previously [37]. All the samples were analyzed using an LSRFortessa flow cytometer (BD Biosciences, Franklin Lakes, NJ, US), and the data were analyzed with FlowJo software. Percentages of immune cells are shown as determined in the CD45^+^ cell population by flow cytometry. The antibodies that were used for staining cells were as follows. Mouse cells: PE-Cy5- or FITC-conjugated anti-mouse CD45 (BD Pharmingen), APC- or PE-Cy7-conjugated anti-mouse CD11b (BD Biosciences), FITC-conjugated anti-mouse Ly6G (BD Biosciences), APC-conjugated anti-mouse G-CSFR (R&D Systems), PE-conjugated anti-mouse CXCR4 (BioLegend), PE-Cy7-conjugated anti-mouse ACKR3 (BioLegend), PerCP-conjugated anti-mouse F4/80 (BioLegend), BV421-conjugated anti-mouse CD11c (BioLegend), PE-conjugated anti-mouse MHC II (BioLegend), FITC-conjugated anti-mouse CD3 (BD Biosciences), APC-conjugated anti-mouse CD4 (BioLegend), BV421-conjugated anti-mouse CD8 (BioLegend), PE-conjugated anti-mouse TCRγδ (BioLegend), and APC-conjugated anti-mouse IgG1 (BioLegend). Human cells: PE-conjugated anti-human G-CSFR (BioLegend) and PE-conjugated anti-human IgG1 (BioLegend). Diverse cell populations were gated as follows: total neutrophils (CD11b^+^ Ly6G^+^), mature neutrophils (CD11b^+^ Ly6G^high^), immature neutrophils (CD11b^+^ Ly6G^low^), DCs (CD11c^+^ MHC II^+^), macrophages (CD11b^+^ F4/80^+^), CD4^+^ T cells (CD3^+^ CD4^+^), CD8^+^ T cells (CD3^+^ CD8^+^), and γδ T cells (CD3^+^ TCRγδ^+^).

### ELISA

The concentrations of G-CSF, IL-3, CXCL12 and CXCL5 in the plasma, bone marrow, or peritoneal lavage fluid of mice were measured using ELISA kits (MCS00, M3000, MCX120, MX000; R&D Systems) according to the manufacturer’s instructions.

### Cell culture, differentiation and transfection

Mouse neutrophils were isolated from the bone marrow, blood or peritoneal cavity of mice as previously described [38]. The cells were resuspended in RPMI-1640 medium supplemented with 10% (v/v) fetal bovine serum (FBS, Gibco) for subsequent experiments or in 1 × PBS for flow cytometric analysis.

The HL-60 cell line was obtained from the American Type Culture Collection (ATCC) and cultured in RPMI-1640 medium supplemented with 10% (v/v) FBS (Gibco) at 37 °C and 5% CO_2_. *ALKBH5*-deficient and WT HL-60 cells were obtained as previously described [38]. For neutrophil differentiation (dHL-60), the medium of HL-60 cells was supplemented with 1 μM all-trans-retinoic acid (ATRA, Sigma) for 4 days. For CSF3R inhibition, the medium containing ATRA was replaced, and the cells were then transfected with siRNA against CSF3R with TransIT-siQUEST Transfection Reagent (Mirus) following the manufacturer’s instructions. Human CSF3R-specific siRNAs were designed and synthesized by GenePharma.

### Bacterial infection and reagents

*E. coli* (JM109 strain, B528410-0001; Sanger Biotech) was grown as previously described [38]. Lipopolysaccharide (LPS) was obtained from *E. coli* O111:B4 (L3024; Sigma). dHL-60 cells were infected with *E. coli* (1 × 10^6^ CFUs), stimulated with LPS (100 ng/ml), or treated with recombinant human G-CSF (100 ng/ml; R&D) for the indicated times.

### RNA-seq

Total RNA was isolated from the indicated bone marrow neutrophils or dHL-60 cells with TRIzol reagent (Invitrogen) and then subjected to Poly(A)+ mRNA purification with a Dynabeads mRNA Purification Kit (61006, Invitrogen) according to the manufacturer’s instructions. RNA samples were quantified with a 2200 Tape Station (Agilent). RNA libraries were prepared with the NEBNext Ultra II Directional RNA Library Prep Kit for Illumina (NEB) according to the manufacturer’s instructions. Two independent biological replicates were performed for RNA-seq.

### Analysis of high-throughput sequencing data

#### General processing

All samples were subjected to paired-end sequencing on the Illumina NovaSeq 6000 platform. Samples were sequenced together in two lanes on one flow cell, and the reads from the two lanes of each sample were combined for analysis. The fastq files were aligned to the reference genome (mm10 or hg38) using HISAT2 (v2.2.1) after removing adapters and low-quality bases. Reads mapped to tRNAs and rRNAs were removed before subsequent analysis.

#### RNA-seq and gene expression analysis

StringTie (v2.1.4) was used to calculate the TPM value of each gene to represent its mRNA expression level. The differentially expressed genes were identified by a negative binomial model using the DEseq2 package by combining information from all replicates. The significantly differentially expressed genes (DEGs) met both of the following criteria: | log_2_ (fold-change)| ≥ 1 and *P* value ≤ 0.05. Gene Ontology biological process enrichment analysis of the DEGs was conducted with the R package clusterProfiler (v3.8.1).

#### m^6^A-seq analysis

m^6^A peak calling was performed as previously described [37]. The longest isoform of each gene was scanned using a 100-bp sliding window with 10-bp steps. We excluded windows with a read count of less than 1/20 of that in the top window in both the input and m^6^A-IP samples to reduce bias from potentially inaccurate gene structure annotations and the arbitrary use of the longest isoform. Sequence motifs on m^6^A peaks were identified and the related *P* values were determined by HOMER.

### RNA extraction and quantitative RT‒PCR

Total RNA was extracted with TRIzol reagent (Invitrogen) or an RNAfast200 Kit (Fastagen). Then, 1 μg of the isolated RNA was reverse transcribed into cDNA with ReverTra Ace qPCR RT Master Mix with gDNA Remover (FSQ-301; TOYOBO), and real-time PCR analysis was then conducted with SYBR Green Real-Time PCR Master Mix (QPK-201; TOYOBO). Amplification products were quantified with a QuantStudio 7 Flex system (Thermo Fisher Scientific). The relative RNA expression levels were normalized to mouse *Gapdh* or human GAPDH according to the ^ΔΔ^C_t_ method. The sequences of the primers used for quantitative RT**‒**PCR are shown in Supplementary Table 1.

### Western blot analysis

These analyses were performed as described previously [37]. Briefly, cells were lysed with RIPA buffer (20-188; Millipore) supplemented with protease inhibitor cocktail and phosphatase inhibitor cocktail (Thermo Fisher Scientific). Then, protein concentrations were measured with a BCA protein assay kit (Thermo Fisher Scientific). Anti-ALKBH5 (HPA007196; Sigma), anti-p-STAT3 (9145T; CST), anti-STAT3 (9139T; CST), anti-G-CSFR (ab156878; Abcam), anti-β-Actin (M117-3; MBL), anti-GAPDH (M171-3; MBL), goat anti-rabbit IgG-HRP (ZB-2301; ZSGB-BIO), and goat anti-mouse IgG-HRP (ZB-2305; ZSGB-BIO) antibodies were used according to the manufacturer’s instructions.

### m^6^A-RIP-qPCR

m^6^A-RIP-qPCR was performed as previously described [37]. In detail, approximately 500 μg of total RNA was extracted with TRIzol reagent (Invitrogen) and then subjected to poly(A)+ mRNA purification with a Dynabeads mRNA Purification Kit (Invitrogen). Purified poly(A)+ mRNA was incubated with an anti-m^6^A antibody (Synaptic Systems) or rabbit immunoglobulin G (IgG, CST) at 4 °C for 2 h and was then immunoprecipitated with Protein A/G beads (Thermo Fisher Scientific) at 4 °C for 2 h. The captured mRNA was competitively eluted with m^6^A nucleotides and then purified by ethanol precipitation. Reverse transcription and qPCR were performed with ReverTra Ace qPCR RT Master Mix with gDNA Remover (TOYOBO) and SYBR Green Real-time PCR Master Mix (TOYOBO) using a QuantStudio 7 Flex system (Thermo Fisher Scientific). The related fold enrichment of m^6^A was calculated by the ^△△^C_t_ method and presented relative to that obtained with IgG. Different regions of human *EEF1A1* mRNA were used as positive and negative controls according to the instructions of the Magna MeRIP m^6^A Kit (Millipore). Primer sequences are listed in Supplementary Table 1.

### RIP-qPCR

RIP-qPCR was performed as previously described [69]. In detail, dHL-60 cells (approximately 3 × 10^7^ cells per sample) were harvested and lysed in IP lysis buffer (Thermo Scientific) and were then incubated with 10 μg of an anti-ALKBH5 antibody (Sigma) or 10 μg of control IgG (Millipore) overnight at 4 °C. Next, the cell lysates were mixed with protein A/G beads (Thermo Scientific) for 2 h at 4 °C. The beads were washed using IP lysis buffer 6 times and were then resuspended in proteinase K to incubate at 56 °C for 1 h. The immunoprecipitated RNAs and input RNAs were isolated using TRIzol reagent for further qRT‒PCR analysis.

### RNA decay assay

*ALKBH5*-deficient and WT dHL-60 cells were seeded at a density of 1 × 10^6^ cells/ml and infected with *E. coli* for 4 h. Then, actinomycin D (5 μg/ml; Sigma) was added to the cell culture medium to block de novo RNA synthesis. After incubation for the indicated times, the cells were harvested, and RNA samples were extracted for qRT‒PCR to measure the levels of *CSF3R* mRNA. The mRNA levels were normalized to those at the *t* = 0 h time point.

### Statistical analysis

The data shown are representative of at least three independent experiments. Mice were randomly assigned to groups for in vivo studies. Data are expressed as the means ± SEMs. All analyses were performed using GraphPad Prism software. Throughout the paper, the sample size (*n*) refers to the number of biological replicates, was determined on the basis of previous experiments using similar methodologies, and is detailed in each figure legend. Statistical significance for the indicated datasets was calculated using two-tailed, unpaired Student’s *t*-test. *P* values of <0.05 were considered to indicate statistical significance.

### Supplementary information

Supplementary Material_clean PDF

Unprocessed original images

## Data Availability

All data are available in the main text or the Supplementary Materials. The current RNA-seq and our previous m^6^A-seq raw data have been deposited in the NCBI Gene Expression Omnibus database under accession numbers GSE224652 (containing two SubSeries, GSE224650 and GSE224651) and GSE201060, respectively.
