# Supplementary material for: The RNA m6A demethylase ALKBH5 drives emergency granulopoiesis and neutrophil mobilization by upregulating G-CSFR expression
Source: Cell Mol Immunol. 2023 Dec 20;21(1):6–18. doi: 10.1038/s41423-023-01115-9 (PMC10757716; doi:10.1038/s41423-023-01115-9)
Supplement: Supplementary file 1 — Supplementary Material_clean PDF [file 41423_2023_1115_MOESM1_ESM.pdf]

Supplementary Materials for

The RNA m^{6}A demethylase ALKBH5 drives emergency granulopoiesis and neutrophil mobilization by upregulating G-CSFR expression

Yang Liu[1,2,3,*], Renjie Song[1], Zhike Lu[4], Lu Zhao[1], Xinyi Zhan[1], Yini Li[4], Xuetao Cao[1,2,3]

Correspondence: Yang Liu (yliu@immunol.org)

This file includes:

Supplementary Figures 1 to 8

Supplementary Table 1

Supplementary Figure 1

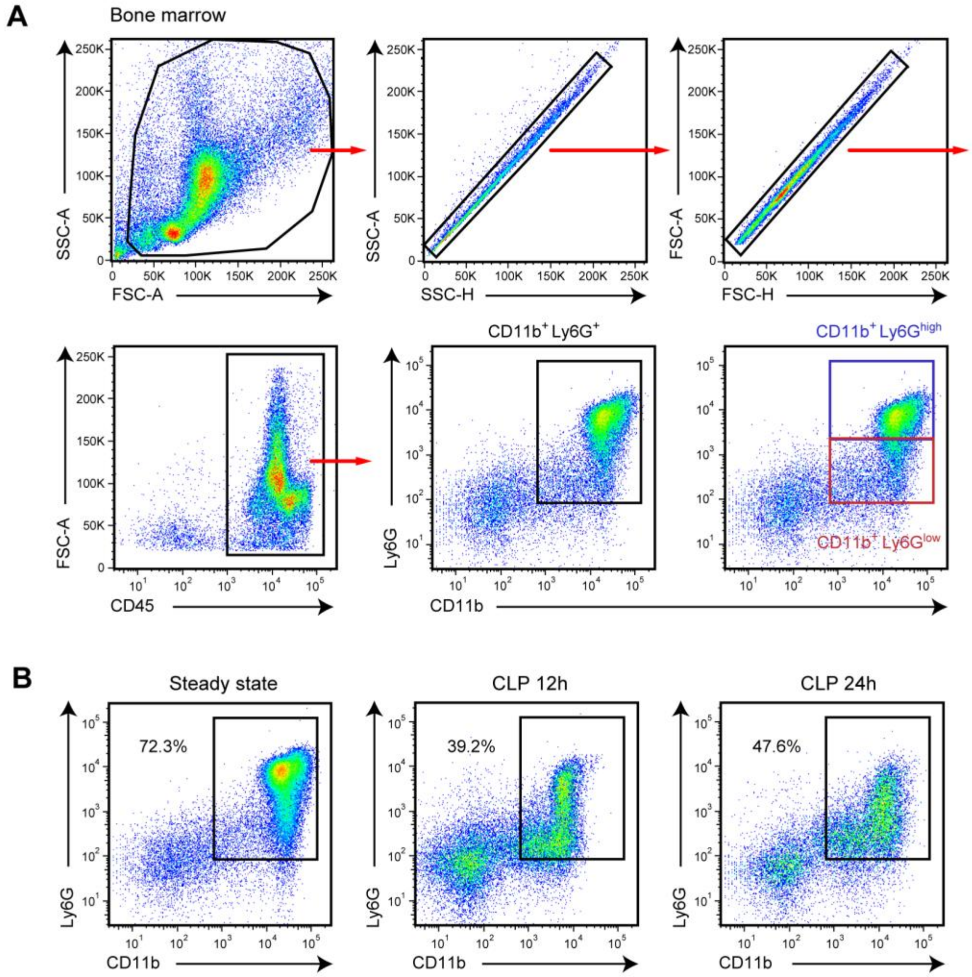

Supplementary Figure 1. Bone marrow neutrophils decrease in septic mice compared to that in steady normal mice. A Gating strategy for analyzing neutrophils in the bone marrow of mice. Black box indicates total neutrophils ($CD11b^{+}$ $Ly6G^{+}$), blue box indicates mature neutrophils ($CD11b^{+}$ $Ly6G^{high}$) and red box indicates immature neutrophils ($CD11b^{+}$ $Ly6G^{low}$), in the same sample. **B** Representative FACS profile showing total neutrophils in bone marrow of wild-type (WT) mice in the steady state or given CLP for indicated times. Related to Fig. 1A. Data are representative of 5 to 10 independent experiments with similar results (A, B).

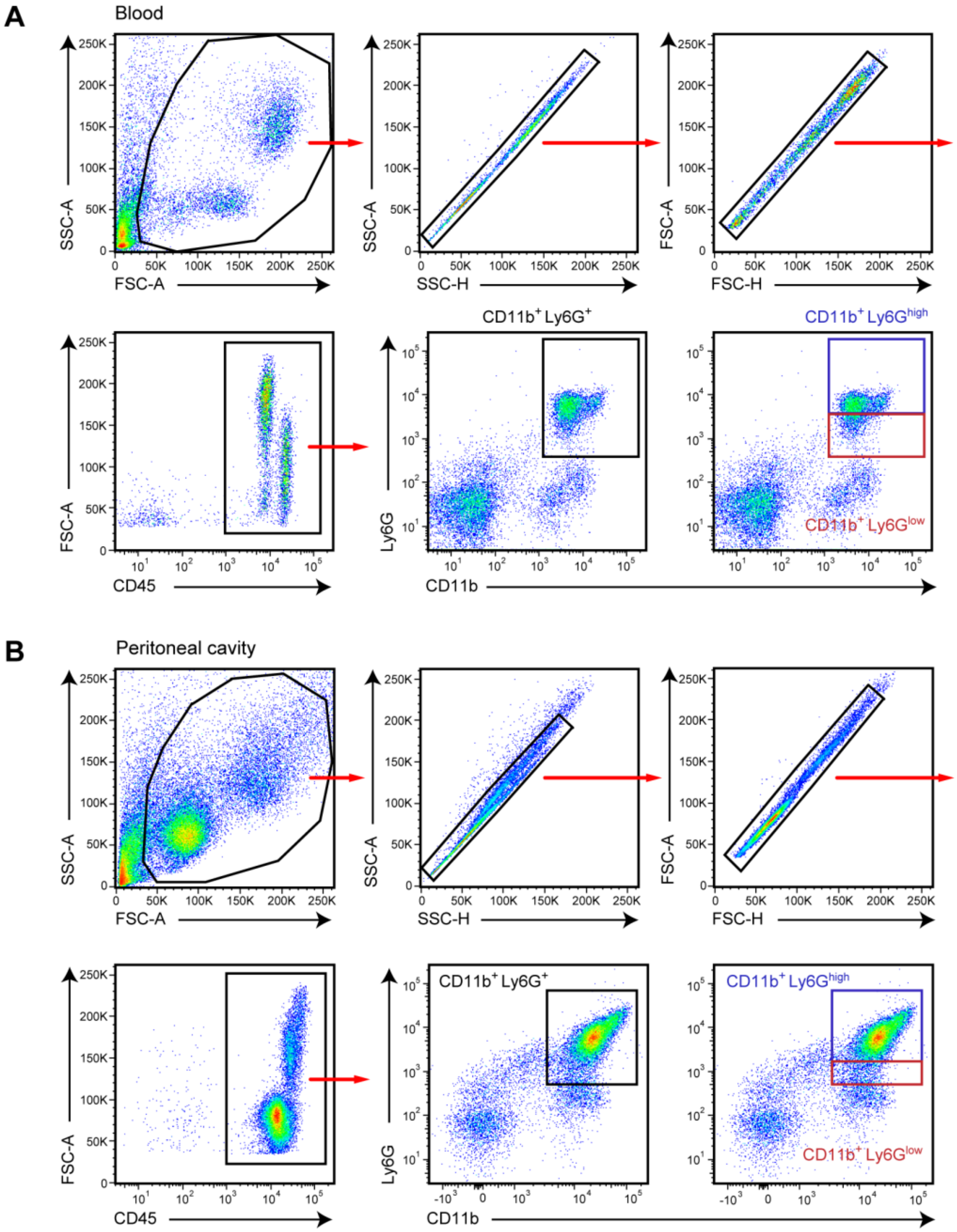

Supplementary Figure 2. FACS analyses of neutrophils in the blood and peritoneal cavity of septic mice. A, B Gating strategies for analyzing neutrophils in the blood (**A**) or peritoneal cavity (**B**) of mice given CLP. Black box indicates total neutrophils (CD11b$^+$ Ly6G$^+$), blue box indicates mature neutrophils (CD11b$^+$ Ly6G^{high}) and red box indicates immature neutrophils (CD11b$^+$

Ly6G^{low}), in the same sample. Related to Fig. 2A-G. Data are representative of 10 or 12 independent experiments with similar results (A, B).

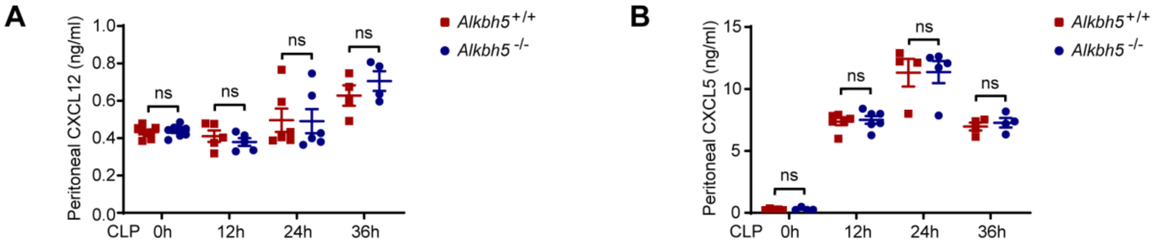

Supplementary Figure 3. ALKBH5 deficiency does not affect the levels of CXCL12 and CXCL5 in peritoneal cavity of mice with or without CLP. A, B Levels of CXCL12 (**A**) and CXCL5 (**B**) determined by ELISA assay in the peritoneal lavage fluid of *Alkbh5*-deficient mice and WT littermates in the steady state or given CLP for indicated times (*n*=4 to 8). All data are mean ± SEM of biologically independent samples. Two-tailed unpaired Student's *t*-test (A, B). ns, not significant.

Supplementary Figure 4

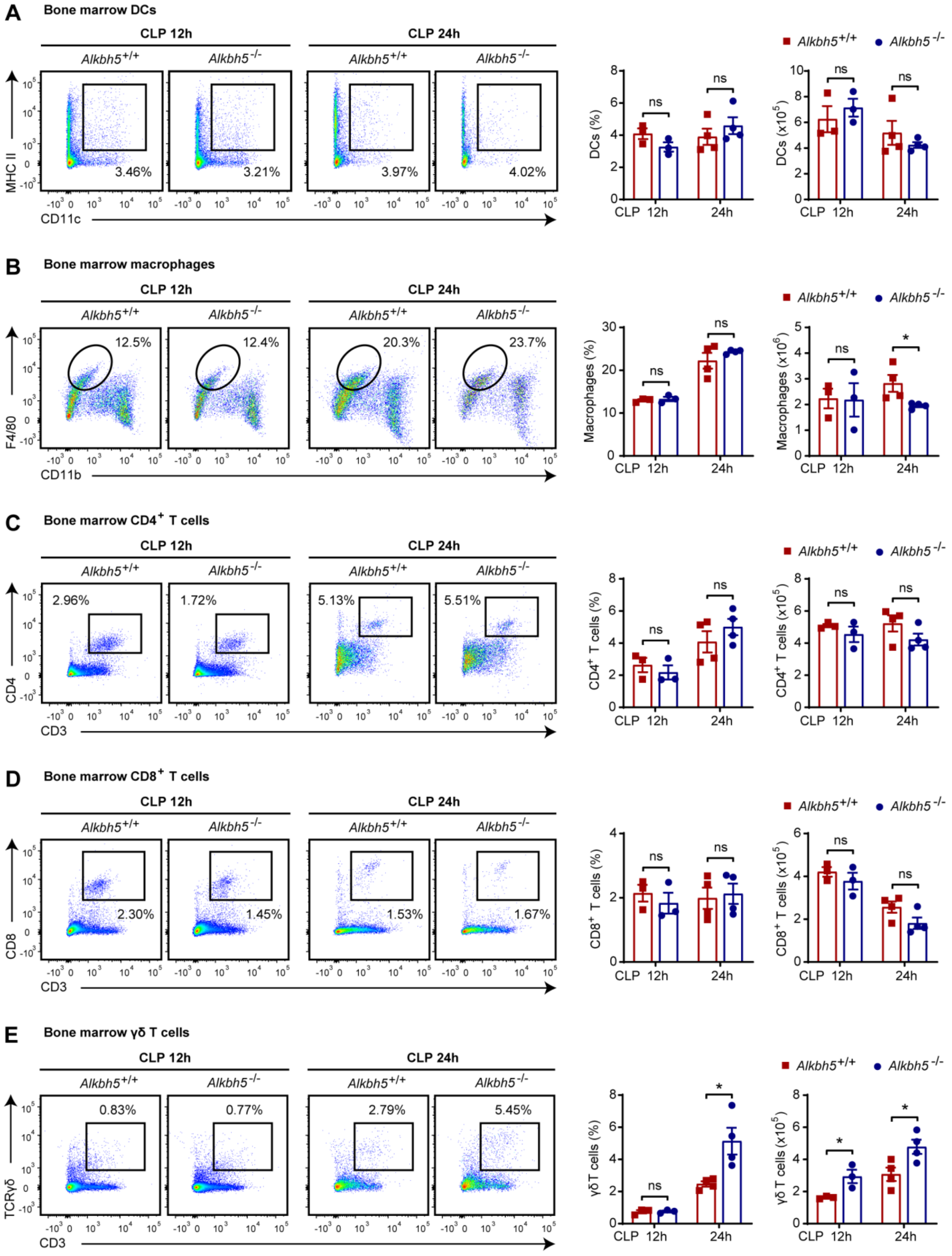

Supplementary Figure 4. Bone marrow leukocyte populations in *Alkbh5*-deficient mice and WT littermates undergoing sepsis. A-E FACS analyses of dendritic cells (DCs, **A**), macrophages (**B**), $CD4^+$ T cells (**C**), $CD8^+$ T cells (**D**) and γδ T cells (**E**) in bone marrow of *Alkbh5*-deficient mice and WT littermates given CLP for 12h or 24h (n=3 or 4). Frequencies in representative FACS profile show the % of $CD45^+$ cells (A-E). All data are mean ± SEM of biologically independent samples. Data are representative of 3 or 4 independent experiments with similar results (A-E). Two-tailed unpaired Student's *t*-test (A-E). *$P < 0.05$; ns, not significant.

Supplementary Figure 5

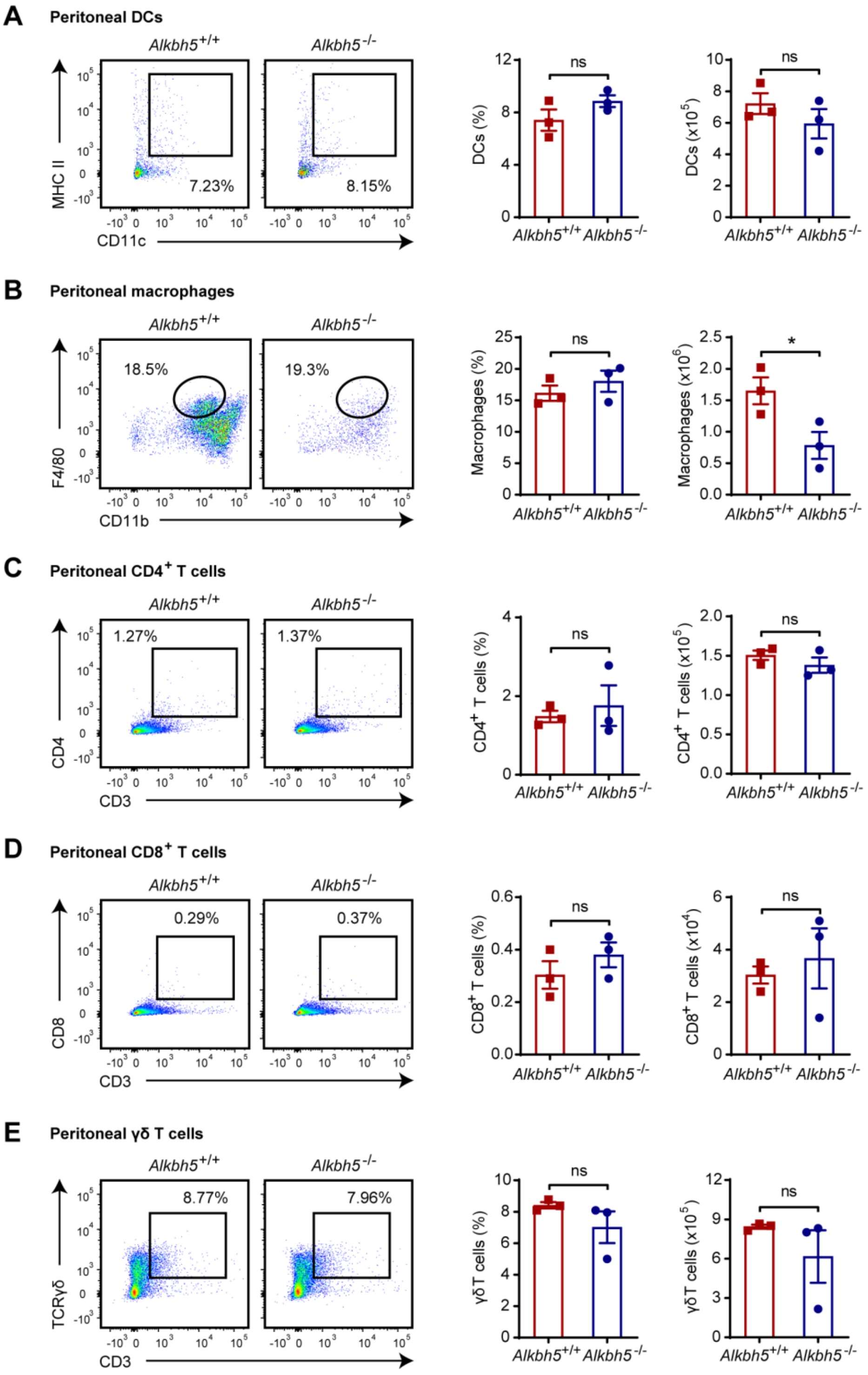

Supplementary Figure 5. ALKBH5 deficiency decreases the numbers of peritoneal macrophages in mice during later stage of sepsis. A-E FACS analyses of dendritic cells (DCs,

A), macrophages (**B**), $CD4^+$ T cells (**C**), $CD8^+$ T cells (**D**) and γδ T cells (**E**) in peritoneal cavity of *Alkbh5*-deficient mice and WT littermates given CLP for 24h (n=3). Frequencies in representative FACS profile show the % of $CD45^+$ cells (A-E). All data are mean ± SEM of biologically independent samples. Data are representative of 3 independent experiments with similar results (A-E). Two-tailed unpaired Student's t-test (A-E). $^*P < 0.05$; ns, not significant.

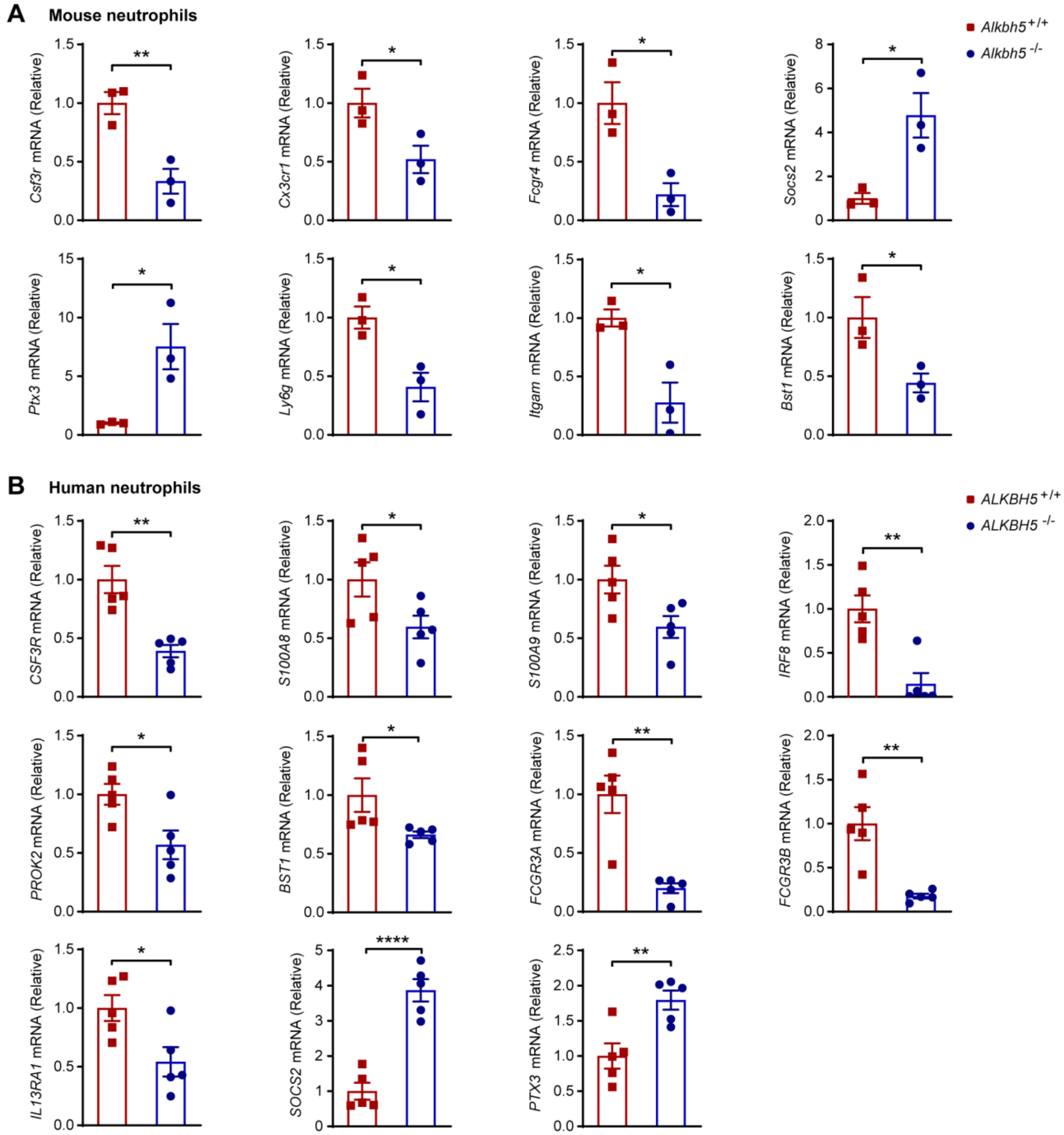

Supplementary Figure 6. ALKBH5 imprints production- and mobilization-promoting gene signatures in both mouse and human neutrophils. A qRT-PCR of the mRNA levels of indicated mouse genes in bone marrow neutrophils isolated from *Alkbh5*-deficient mice and WT littermates given CLP (*n*=3). **B** qRT-PCR of the mRNA levels of indicated human genes in *ALKBH5*-deficient

and WT dHL-60 cells with *E.coli* infection (*n*=5). qRT-PCR data were normalized to *Gapdh* (A) or *GAPDH* (B) expression. All data are mean ± SEM of biologically independent samples. Two-tailed unpaired Student's *t*-test (A, B). **P* < 0.05; ***P* < 0.01; *****P* < 0.0001.

Supplementary Figure 7

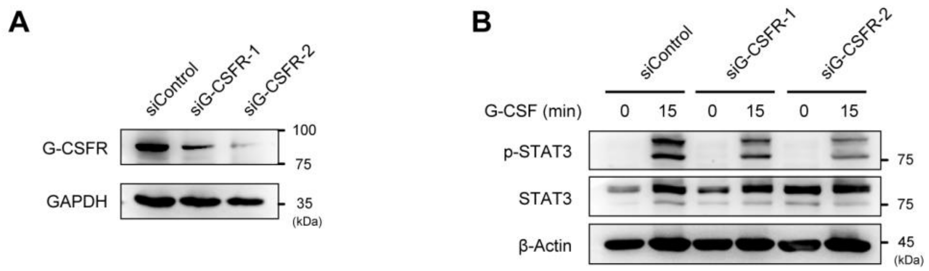

Supplementary Figure 7. Knockdown of G-CSFR suppresses G-CSF-induced STAT3 activation in neutrophils. A Immunoblot analysis of G-CSFR levels in dHL-60 cells transfected with the control and two independent G-CSFR siRNAs for 48h. **B** Immunoblot analysis of STAT3 signaling in dHL-60 cells transfected with indicated siRNAs and then treated with human G-CSF (100ng/ml) for indicated times. Data are representative of 3 independent experiments with similar results (A, B).

Supplementary Figure 8

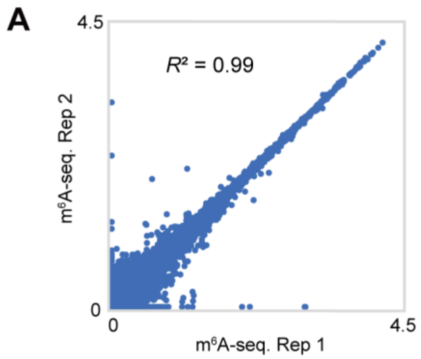

Supplementary Figure 8. Strong correlation between two biological replicates of m^{6}A-seq. A Scatter plots showing a strong correlation between two biological replicates of our m^{6}A-seq (GSE201060). The R-squared value (R^2) was shown as indicated.

Supplementary Table 1. Primer sequences used for qRT-PCR.

Primers for mouse genes	Sequence (5′→3′)
Csf3r Forward	TGGACACATCGAGATTTCACCC
Csf3r Reverse	GGTGTAGTTCAAGTGAGGCAG
Cx3cr1 Forward	GAGTATGACGATTCTGCTGAGG
Cx3cr1 Reverse	CAGACCGAACGTGAAGACGAG
Fcgr4 Forward	AAAGTCCTGTGGCTGACTATG
Fcgr4 Reverse	GCTGCTGCTTTGCTCTAATG
Socs2 Forward	AGTTCGCATTCAGACTACCTACT
Socs2 Reverse	TGGTACTCAATCCGCAGGTTAG
Ptx3 Forward	CGCAGGTTGTGAAACAGCAAT
Ptx3 Reverse	GGGTTCCACTTTGTGCCATAAG
Ly6g Forward	TTCCTGCAACACAACTACC
Ly6g Reverse	GATGGGAAGGCAGAGATTG
Itgam Forward	ATGGACGCTGATGGCAATACC
Itgam Reverse	TCCCCATTCACGTCTCCCA
Bst1 Forward	AGGGACAAGTCACTGTTCTGG
Bst1 Reverse	AACTTTGCCATACAGCACGTC
Gapdh Forward	AGGTCGGTGTGAACGGATTTG
Gapdh Reverse	TGTAGACCATGTAGTTGAGGTCA
**Primers for human genes**	**Sequence (5′→3′)**
CSF3R Forward	GAGCTGAGAACTACCGAACGG
CSF3R Reverse	GGCCTGAGGGTCTCCAAGA
S100A8 Forward	ATGCCGTCTACAGGGATGAC
S100A8 Reverse	ACGCCCATCTTTATCACCAG
S100A9 Forward	GAATTCAAAGAGCTGGTGCGA
S100A9 Reverse	GCTTGTCTGCATTTGTGTCCA
IRF8 Forward	GTCTTCGACACCAGCCAGTT
IRF8 Reverse	AGCTCTTCCCAGCCTCTTCT
PROK2 Forward	TGTGACAAGGACTCCCAATGTGGTG
PROK2 Reverse	CCGCCCAAAAAATGGAACTTTACG
BST1 Forward	ACAGCACCCATCCTGACTGT
BST1 Reverse	GAAGCCAGCACCAGAAAGAG
FCGR3A Forward	CCTCCTGTCTAGTCGGTTTGG
FCGR3A Reverse	TCGAGCACCCTGTACCATTGA
FCGR3B Forward	CCTCAATGGTACAGCGTGCTT

FCGR3B Reverse	TATGATGAAAATACTTCCTGTCTT
IL13RA1 Forward	GTGCCTTTAACTTCCCGTGT
IL13RA1 Reverse	CCCATTGCACATATAGGTCATC
SOCS2 Forward	TGCAAGGATAAGCGGACAGG
SOCS2 Reverse	CAGAGATGGTGCTGACGTGT
PTX3 Forward	GCTCTCTGGTCTGCAGTGTT
PTX3 Reverse	GGTCCTCAGTGGGATGGAGT
EEF1A1 Positive Forward	CGGTCTCAGAACTGTTTGTTTC
EEF1A1 Positive Reverse	AAACCAAAGTGGTCCACAAA
EEF1A1 Negative Forward	GGATGGAAAGTCACCCGTAAG
EEF1A1 Negative Reverse	TTGTCAGTTGGACGAGTTGG
GAPDH Forward	TGCACCACCAACTGCTTAGC
GAPDH Reverse	GGCATGGACTGTGGTCATGAG
18S rRNA Forward	GTAACCCGTTGAACCCCATT
18S rRNA Reverse	CCATCCAATCGGTAGTAGCG
