# Supplementary material for: The RNA m6A demethylase ALKBH5 drives emergency granulopoiesis and neutrophil mobilization by upregulating G-CSFR expression
Source: Cell Mol Immunol. 2023 Dec 20;21(1):6–18. doi: 10.1038/s41423-023-01115-9 (PMC10757716; doi:10.1038/s41423-023-01115-9)

Supplementary Information of unprocessed original images

Figure 4D

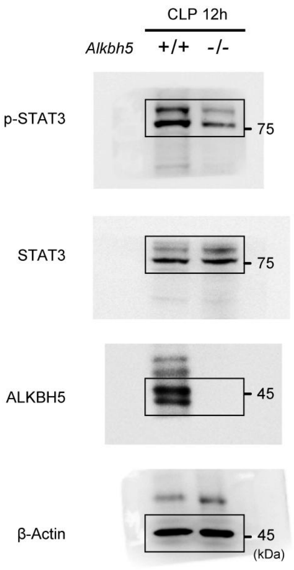

Figure 4E

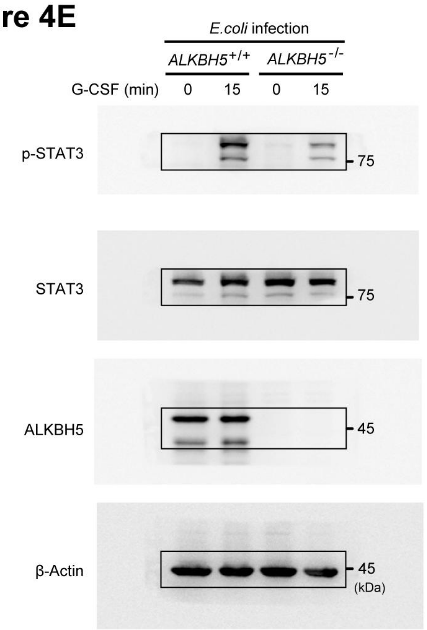

Supplementary Figure 7A

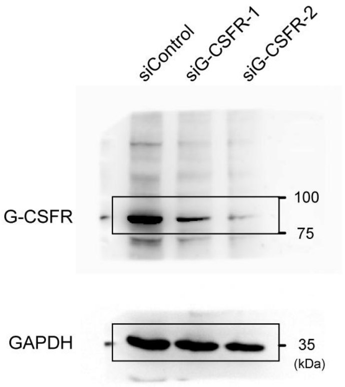

Supplementary Figure 7B

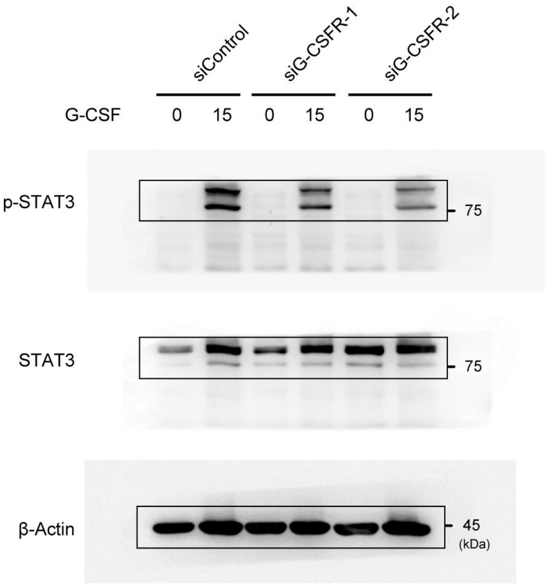

Supplement: Supplementary file 2 — Unprocessed original images [file 41423_2023_1115_MOESM2_ESM.pdf]
